# Phylogenetics, Niche Evolution, and Distribution Dynamics of *Isatis* Species Under Climate Change

**DOI:** 10.1002/ece3.73514

**Published:** 2026-05-04

**Authors:** Min Wei, Yong Su, Hongzhuan Shi, Tao Bao, Qiaosheng Guo

**Affiliations:** ^1^ Institute of Chinese Medicinal Materials Nanjing Agricultural University Nanjing Jiangsu People's Republic of China; ^2^ China Resources Sanjiu Medical & Pharmaceutical Co., Ltd Shenzhen People's Republic of China; ^3^ Shenzhen Traditional Chinese Medicine Manufacturing Innovation Center Co., Ltd. Shenzhen People's Republic of China; ^4^ Key Laboratory of Horticultural Crop Germplasm Innovation and Utilization (Coconstruction by Ministry and Province), Institute of Horticulture Anhui Academy of Agricultural Sciences Hefei China

**Keywords:** climate change, ecological strategies, *Isatis*, MaxEnt model, niche conservatism

## Abstract

*Isatis* is a vital medicinal genus within the Brassicaceae family, yet key knowledge gaps persist regarding its environmental drivers, niche evolution patterns, and climate change responses—hindering the conservation and utilization of its germplasm resources. To address these, this study integrated 229 occurrence records of six *Isatis* species and 144 environmental variables. Using an optimized MaxEnt model, we predicted their potential geographic distributions under current and future climate scenarios (SSP245 and SSP585) and analyzed their niche evolution patterns in a phylogenetic context. The results revealed that: (1) excellent model performance (all AUC > 0.92); (2) clear life‐history‐based ecological differentiation—annual short‐lived plants (*I. violascens*, *I. minima* ) were primarily constrained by precipitation seasonality, whereas biennial species (cultivated *I. indigotica*, wild *I. costata* ) were more strongly associated with habitat indicators such as growing‐season vegetation phenology; (3) complex niche evolution patterns rejecting strict conservatism—niche conservatism in closely related pairs (*I. indigotica* and *I. costata* ) coexisting with niche convergence in distantly related annual ephemerals (*I. violascens* and *I. minima* ); and (4) divergent future trajectories—suitable habitat for *I. indigotica* remains stable, while that for *I. costata* is projected to contract by ~18.5%. This study elucidates adaptive evolution in *Isatis* through the lens of life‐history strategies and niche evolution, highlights the elevated extinction risk faced by wild relatives, and provides a scientific foundation for targeted conservation and sustainable utilization of this genus' germplasm resources.

## Graphical abstract

This study systematically elucidates the adaptive evolution of *Isatis* species from the perspective of life‐history strategies and niche evolution, highlights the increased extinction risk faced by wild relatives, and provides a critical scientific basis for the conservation and sustainable use of the germplasm resources of this genus.

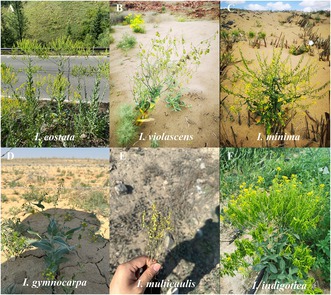

## 1 Introduction

Global climate change and anthropogenic activities are altering the geographical distributions of species at unprecedented scales and rates, posing severe challenges to biodiversity conservation and the stability of ecosystem functions (Urban et al. 2024; Spencer et al. 2024). In this context, accurately predicting species' potential distributions, deciphering their ecological requirements, and assessing their climatic vulnerability are of paramount importance for planning conservation and sustainable resource utilization (Rinas and Vellend 2024). Species distribution models (SDMs) have consequently emerged as a core tool to address this challenge (Vasconcelos et al. 2024). Medicinal plants, as key components of biodiversity, demand particular attention because their quality and survival are directly linked to human health and well‐being (Tomou et al. 2022; Theodoridis et al. 2023).

The genus *Isatis* Tourn. ex L. (Brassicaceae) comprises approximately 85–92 species distributed continuously from the Mediterranean basin across the Irano‐Turanian region to eastern Asia (Al‐Shehbaz 2025; Royal Botanic Gardens Kew, n.d.‐a). This broad Eurasian distribution spans a remarkable ecological gradient—from the winter‐wet Mediterranean woodlands and steppes, through the vast arid and semi‐arid deserts of Central Asia, to the montane forests and agricultural landscapes of eastern Asia. Throughout this range, many *Isatis* species possess significant medicinal and economic value (Han et al. 2022; Speranza et al. 2020; Fozia et al. 2022). The most notable is *Isatis indigotica* Fortune ex Lindl., which has been valued for centuries both as a source of blue dye and as a traditional medicinal herb in the Chinese Pharmacopeia (Committee N P 2025). We follow the Pharmacopeia of China in using the name *I. indigotica*, while acknowledging that it is treated as a synonym of *Isatis tinctoria* L. in global databases such as Plants of the World Online (POWO); recent genomic evidence supports their recognition as independently domesticated lineages that diverged approximately 6.7 million years ago (Su et al. 2023; Chen et al. 2025).

Within this broad Eurasian continuum, China—particularly the Xinjiang region—represents the eastern extent of the genus' natural distribution and harbors a representative assemblage of species exhibiting diverse life‐history strategies. These range from annual desert ephemerals (e.g., *Isatis minima* Bunge, *Isatis violascens* Bunge) adapted to the extreme aridity of the Junggar Basin, to biennial species occupying more mesic habitats such as forest edges and river valleys (*Isatis costata* C. A. Mey.), and the widely cultivated *I. indigotica* which thrives in agricultural systems (Figure S5) (Alexeyev 2014; Peng, He, Jiang, et al. 2023; Lu et al. 2015). This “cultivated‐wild” complex within a defined geographic area provides a unique opportunity to investigate how contrasting ecological strategies shape species' responses to environmental drivers. The ecological diversity of these six species is summarized in Box 1.

BOX 1 **Ecological diversity of Chinese *Isatis* species.**

**Table:** 

Species	Life history	Habitat	Distribution	Phylogenetic placement
*I. indigotica*	Biennial (cultivated)	Farmland	Cultivated in China, mainly in northern regions; no wild populations known	Clade A (with *I. costata* )
*I. costata*	Biennial (wild)	Mountain slopes, forest edges, river valleys	Inner Mongolia, Xinjiang, Liaoning, Gansu	Clade A
*I. minima*	Annual (ephemeral)	Semi‐fixed sand dunes	Gurbantunggut Desert, Xinjiang; also Gansu	Clade B
*Isatis multicaulis* (Kar. & Kir.) Jafri	Annual (ephemeral)	Gravelly sandy land	Xinjiang, Gansu	Clade C
*Isatis gymnocarpa* (Fisch. ex DC.) Al‐Shehbaz, Moazzeni & Mumm.	Annual (ephemeral)	Gravelly sandy land	Xinjiang, Tibet	Clade D (with *I. violascens*)
*I. violascens*	Annual (ephemeral)	Semi‐fixed sand dunes	Gurbantunggut Desert, Xinjiang	Clade D

*Note:* Phylogenetic placement based on chloroplast genome data (Wei et al. 2026). These clades are sequentially diverged: Clade D is the earliest diverging lineage, followed by Clade C, then Clade B, and finally Clade A as the most recent.

Despite this ecological diversity, fundamental questions remain unanswered. The contrasting life histories (biennial vs. annual) and phylogenetic relationships (Clades A–D) summarized in Box 1 provide a natural framework for testing two specific hypotheses. First, if life‐history strategy shapes environmental sensitivity, then annual ephemerals and biennials should be limited by different environmental drivers. Second, if niche evolution follows phylogenetic constraints, closely related species should show high niche overlap; alternatively, if strong selection overrides history, distantly related species occupying similar habitats should show convergent niches. Within the established phylogenetic context, what core environmental factors drive the distributions of cultivated *I. indigotica* and its wild relatives? Have they evolved distinct ecological adaptation strategies? Does niche evolution within *Isatis* conform with the classic hypothesis of niche conservatism (Riera et al. 2025)? In the face of ongoing climate change, what risks will these species, which hold significant economic and genetic value, face in terms of their survival space? To date, no systematic and quantitative assessment addressing these questions has been conducted across their Chinese range (Lan et al. 2022).

To address these knowledge gaps, we selected six *Isatis* species that collectively represent the full spectrum of ecological strategies and phylogenetic lineages within China. These include the cultivated biennial *I. indigotica*, its wild biennial relative *I. costata* , and four annual desert ephemerals (*I. violascens*, *I. minima* , *I. multicaulis*, *I. gymnocarpa*) (Wu et al. 2001). This selection allows us to compare niche evolution and climate vulnerability across contrasting life histories (annual vs. biennial) and evolutionary distances (closely related vs. distantly related pairs). Using an integrated approach combining optimized MaxEnt models, niche analysis, and phylogenetic comparisons, this study aimed to: (1) predict the current potential geographic distributions of these species; (2) identify the core environmental drivers and ecological strategy types; (3) test the niche conservatism hypothesis within the genus; and (4) project future shifts in suitable habitats under climate change scenarios to assess climatic risks.

This study is expected to deepen our understanding of the adaptive evolution of *Isatis* species from the perspectives of ecological strategy divergence and niche evolution while also providing a novel case study for exploring the mechanisms underlying plant distribution patterns. These findings provide a critical scientific basis for the effective conservation of *Isatis* germplasm resources, rational planning for its cultivation, and the development of adaptive strategies to mitigate the effects of climate change.

## 2 Materials and Methods

### 2.1 Species Occurrence Data

In this study, 261 geographic occurrence records for six *Isatis* species were initially compiled. The data were sourced primarily from multiple sources: field surveys conducted between 2021 and 2023 (for wild species), direct communication with local farmers (for the cultivated *I. indigotica*), and supplemented by records from the Chinese Virtual Herbarium (CVH) (Chinese V H, n.d.), the Global Biodiversity Information Facility (GBIF) (Royal Botanic Gardens Kew, n.d.‐b), the National Specimen Information Infrastructure (NSII) (NSII, n.d.), and related literature.

**FIGURE 1:**
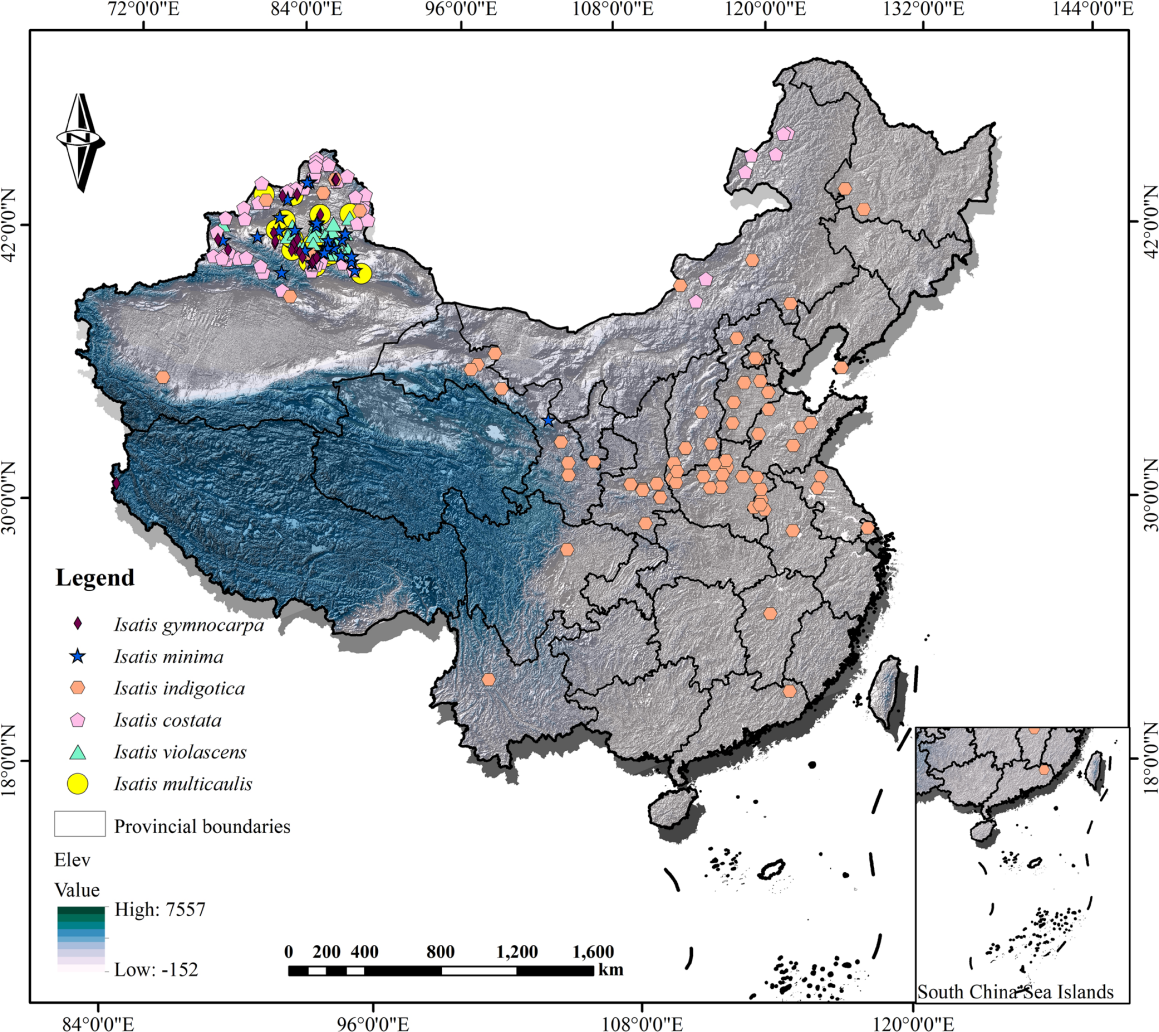
Distribution of six *Isatis* species after screening. Points are colored by species. The basemap was obtained from the Standard Map Service of the Ministry of Natural Resources, China (Approval No.: GS (2023) 2767).

During the data preprocessing stage, all occurrence records underwent rigorous cleaning. Records with missing or clearly erroneous coordinate information, as well as completely duplicate records, were removed. To mitigate spatial autocorrelation, occurrence points were spatially rarefied in ENMTools to one point per 1 km^2^ grid cell. Following this process, 229 unique records were retained for final modeling (*I. indigotica*: 70; *I. costata* : 60; *I. violascens*: 31; *I. minima* : 27; *I. gymnocarpa*: 24; *I. multicaulis*: 17; see Table S1). Among the 70 *I. indigotica* records, two points in Yunnan and western Xinjiang represent small‐scale cultivated populations outside the main production areas; these were retained as valid reflections of the species' full cultivation range. We verified that all 229 retained records had complete data for all 144 environmental variables within the study area; therefore, no records were excluded due to missing environmental data. The final sample sizes thus reflect true sampling effort and species rarity rather than data gaps. Specifically, the four annual wild species with modest sample sizes are narrow‐ranged taxa with naturally restricted distributions (see Box 1), and no additional populations were identified during extensive field surveys across their potential habitats. The spatial distribution of these filtered records is shown in Figure 1. All final occurrence points were formatted as CSV files for use in MaxEnt version 3.4.4. The basemap of China used in this study was obtained from the Standard Map Service of the Ministry of Natural Resources (Approval No.: GS (2023) 2767). All environmental variable layers were masked to this boundary and clipped using ArcGIS 10.8, with the extent of the analysis uniformly limited to the region of China.

### 2.2 Environmental Variables and Screening

#### 2.2.1 Data Sources

A total of 144 environmental variables across six categories were compiled to characterize habitat conditions influencing species distributions (Table 1). All variables were standardized to a spatial resolution of 30 arc‐seconds (approximately 1 km). The specific data sources are as follows: (1) Climate Data: Nineteen bioclimatic variables for the period 1970–2000 were obtained from the WorldClim database (Fick and Hijmans 2017). (2) Meteorological Data: Monthly data for the year 2020 were acquired from the Chinese Meteorological Element Spatial Interpolation Dataset (Xu 2022). (3) Soil Data: Physicochemical properties such as soil texture and organic carbon content were sourced from the Harmonized World Soil Database (HWSD) (FAO A I 2023). (4) Topographic Data: A digital elevation model (DEM) and derived slope and aspect data were obtained from the USGS EROS Center (Farr et al. 2007). (5) Vegetation Data: Monthly and annual normalized difference vegetation index (NDVI) data for 2020 were downloaded from the NASA Earthdata platform (Didan 2015). (6) Irrigated Cropland Data: The distribution data of irrigated cropland at a 250‐m resolution for 2020 were sourced from the Chinese Irrigation Cropland Distribution Dataset (Zhang et al. 2024). Detailed information for all 144 initial variables is provided in Table S2, and specific versions and references for the core data sources are detailed in the Supporting Information.

**TABLE 1:** Data types and sources of environmental variables.

Data type	Source	Representative variables	Number of variables
Bioclimatic	WorldClim v2.1	Annual Mean Temperature, Annual Precipitation, etc.	19
Monthly meteorological	Chinese Meteorological Element Dataset	Monthly Evaporation, Monthly Precipitation, etc.	96
Soil properties	HWSD v2.0	Soil pH, Organic Carbon Content, etc.	10
Topography	SRTM	Elevation, Slope, Aspect, etc.	5
Vegetation indices	NASA MOD13A3	Annual and Monthly NDVI, etc.	13
Irrigated cropland	Chinese Irrigation Cropland Distribution Dataset	CIrrMap250	1

*Note:* This table summarizes the data sources, representative variables, and number of variables across the six categories used for constructing the species distribution models. All the variables were uniformly resampled to a spatial resolution of approximately 1 km. The bioclimatic variables represent the long‐term climatic normals for 1970–2000, whereas the monthly meteorological, vegetation index, and irrigated cropland data correspond to the year 2020. The complete list and detailed descriptions of all 144 initial variables are provided in Table S2.

#### 2.2.2 Variable Screening

The initial pool of 144 environmental variables was assembled to capture the full range of ecological factors potentially shaping *Isatis* distributions, reflecting the genus' diverse life‐history strategies. For desert ephemerals, monthly climatic data were included to capture critical phenological windows—such as early spring temperature and moisture conditions—that regulate seed germination and early survival, yet are often masked by annual averages (Zhou et al. 2015; Lu et al. 2017). Soil properties were incorporated because soil texture and nutrient availability influence root architecture, metabolite profiles, and root‐associated fungal communities in desert ephemerals (Peng, He, Jiang, et al. 2023; Peng, He, Wang, et al. 2023). Topographic variables (elevation, slope, aspect) were included to account for water and heat redistribution, as field surveys confirmed species' distinct distributional patterns along elevational gradients. Vegetation indices and irrigated cropland proportion were additionally considered to capture biotic interactions and anthropogenic influences, given that cultivation practices affect soil microbial activity and plant performance in *I. indigotica* (Liu and Tan 2007; He and Wang 2021).

To reduce overfitting and multicollinearity while retaining ecologically meaningful predictors, we applied a three‐step variable screening procedure for each species: (1) Category‐specific correlation‐based screening: To avoid redundancy within each variable type, we performed Pearson correlation analyses separately for climatic, monthly meteorological, soil, and vegetation variables. For any pair of variables within the same category with |*r*| > 0.8, we retained the variable with higher ecological interpretability based on prior knowledge of the species' ecological traits (Box 1). A sensitivity analysis using a more conservative threshold (|*r*| > 0.7) confirmed that the core environmental drivers remained consistent. (2) Contribution‐based selection: Using all occurrence points and the candidate variables, we ran preliminary MaxEnt models and ranked variables by their mean percent contribution. For each species, we retained 22–23 variables that best captured the major environmental signals. (3) Post‐selection correlation check: We then examined pairwise Pearson correlations among the retained variables (Figure S1). All variables listed in Table S3 were included in the final models; their percent contributions and permutation importances are reported therein.

### 2.3 Species Distribution Modeling: Parameter Optimization, Execution, and Evaluation

We employed MaxEnt for species distribution modeling due to its suitability for our dataset and research objectives. MaxEnt performs robustly with small sample sizes—a critical advantage given that four wild relatives in this study have only 17–31 records (Merow et al. 2013; van Proosdij et al. 2016)—while its continuous habitat suitability outputs support subsequent analyses (niche overlap, species richness mapping, range shift quantification), and its variable importance rankings and response curves directly address the environmental drivers underlying contrasting ecological strategies between annual ephemerals and biennials. To mitigate model overfitting and variable correlation, we adopted a stratified parameter optimization strategy tailored to the sample sizes of different species groups.

For cultivated species *I. indigotica* and its wild relative *I. costata* , which have relatively larger sample sizes (70 and 60 records, respectively) and are central to our comparative analyses, we performed systematic parameter tuning using the ENMeval R package (Muscarella et al. 2014). The tested feature combinations (FCs) included H, L, LQ, LQH, LQHP, and LQHPT. The regularization multiplier (RM) was tested across a range from 0.5 to 4, with an increment of 0.5. The Akaike information criterion corrected for small sample sizes (AIC_c_) was calculated, and the model with the smallest delta AIC_c_ score was selected as the final optimized model. This procedure was designed to provide a high‐precision predictive foundation for subsequent in‐depth comparisons of their niches and future projections. For the remaining four wild relatives, which have limited sample sizes, we employed the widely validated default MaxEnt parameters following established recommendations for small‐sample modeling.

All the models were run with the following unified settings: 75% of the occurrence data were used for training and 25% for testing, the bootstrap method was applied with 10 replicates, and the output format was set to “Cloglog”. Model predictive performance was evaluated using the area under the receiver operating characteristic curve (AUC). To quantify the extent of suitable habitats, the continuous habitat suitability raster (0–1) output from the models was reclassified into four grades using the natural breaks (Jenks) method: nonsuitable area (0 < *p* ≤ 0.2), low‐suitability area (0.2 < *p* ≤ 0.4), medium‐suitability area (0.4 < *p* ≤ 0.6), and high‐suitability area (0.6 < *p* ≤ 1) (Liu et al. 2025; Shi 2023). These thresholds are statistically derived boundaries for visualization purposes only and do not represent absolute biological cutoffs between presence and absence; all continuous predictions remain available in the model outputs. In subsequent analyses, we focused on variables with permutation importance ≥ 10% in at least one species—a threshold commonly used in SDM studies to highlight core drivers without excluding other variables from the models (Thuiller et al. 2019; Bellard et al. 2013).

### 2.4 Biogeographic and Evolutionary Ecological Analyses

#### 2.4.1 Species Richness and Hotspot Mapping

To reveal the macroscale distribution patterns at the genus level, we generated a total species richness map by spatially overlaying the binarized suitable habitat maps of all six species, aiming to identify the distributional hotspots of *Isatis* in China. Furthermore, to investigate the distribution patterns of specific groups with distinct ecological strategies, we created additional richness maps for two species pairs: “*I. indigotica*– *I. costata* ” and “*I. violascens*– *I. minima* ”. All overlay and mapping procedures were performed using ArcGIS 10.8 software.

#### 2.4.2 Niche Evolution Analysis (Mantel Test)

A Mantel test was performed to test the niche conservatism hypothesis within the *Isatis* genus, which posits that closely related species tend to possess more similar ecological niches. The phylogenetic tree (Figure S2) and branch length data were derived from our previously published study, which was constructed on the basis of complete chloroplast genomes (Wei et al. 2026). The pairwise branch length distances for all 15 species pairs were calculated to form the phylogenetic distance matrix. On the basis of the continuous habitat suitability maps generated by the MaxEnt model for each species under current climate conditions, Schoener's *D* index was computed for all 15 species pairs to form the niche overlap matrix (Warren et al. 2008). The Mantel test was executed using the vegan package in R, with 9999 permutations, to calculate the correlation coefficient (*r*) and its significance (*p* value). The complete data on genetic distances and niche overlap (Schoener's *D*) for all 15 species pairs are provided in Table S4.

#### 2.4.3 Future Climate Change Scenario Analysis

To assess the potential impacts of future climate change on *Isatis* species, we utilized the BCC‐CSM2‐MR model from CMIP6 to project the potential geographical distributions of all six species for the period 2041–2060 under two scenarios, SSP245 and SSP585 (Howarth and Viner 2022). Future continuous habitat suitability maps and binary distribution maps were generated for each. To gain a deeper understanding of how species with different ecological strategies respond to climate change, we focused our spatiotemporal change analysis on cultivated *I. indigotica* and its wild relative *I. costata* , as they present distinct environmental driving patterns. By comparing the spatial shifts and area dynamics of their current and future suitable habitats, we aimed to elucidate their potential differential response mechanisms.

## 3 Results

### 3.1 Model Performance and Parameter Optimization

The MaxEnt models demonstrated excellent predictive performance for all six *Isatis* species, with the mean test AUC values all exceeding 0.92 (Table 2). *I. gymnocarpa* achieved the highest test AUC (0.995), indicating near‐perfect model discrimination.

**TABLE 2:** Parameters and performance evaluation of MaxEnt models for *Isatis* species.

Species	Modeling approach	FC	RM	Training AUC	Test AUC
*I. indigotica*	Parameter‐tuned	LQ	4.0	0.996	0.956
*I. costata*	Parameter‐tuned	L	3.5	0.991	0.967
*I. violascens*	Default parameter	LQHP	1	0.99	0.991
*I. minima*	Default parameter	LQHP	1	0.997	0.929
*I. gymnocarpa*	Default parameter	LQHP	1	0.963	0.995
*I. multicaulis*	Default parameter	LQHP	1	0.974	0.984

*Note:* Models for *I. indigotica* and *I. costata* were built with parameters optimized using ENMeval, while models for the remaining species employed the default MaxEnt parameters. The AUC values represent the mean of 10 bootstrap replicates.

**FIGURE 2:**
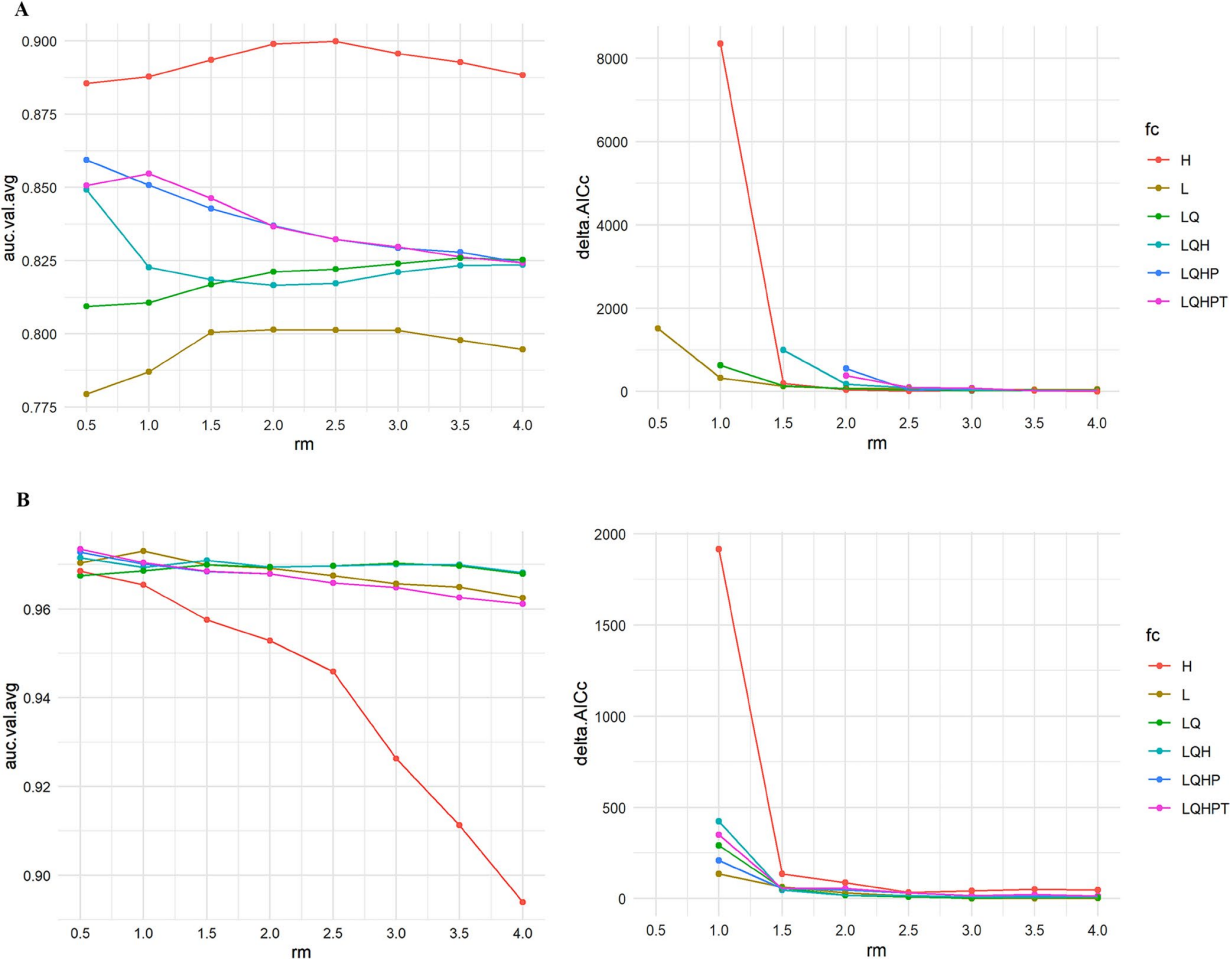
Parameter optimization of MaxEnt models for *I. indigotica* and *I. costata* . Panel (A) shows the parameter optimization results for *I. indigotica*, and Panel (B) shows those for *I. costata* .

The parameter tuning process for *I. indigotica* and *I. costata* revealed distinct optimal model complexities for each species (Figure 2). The model for *I. indigotica* achieved the lowest AIC_c_ score, with a linear and quadratic feature combination (FC = LQ) and a regularization multiplier of 4.0 (RM = 4.0). In contrast, the optimal model for *I. costata* used only linear features (FC = L) with an RM of 3.5. These optimized models also exhibited low training omission rates (3.6% and 2.2%, respectively), confirming a good fit to the training data.

### 3.2 Dominant Environmental Drivers and Ecological Differentiation of *Isatis* Species

**FIGURE 3:**
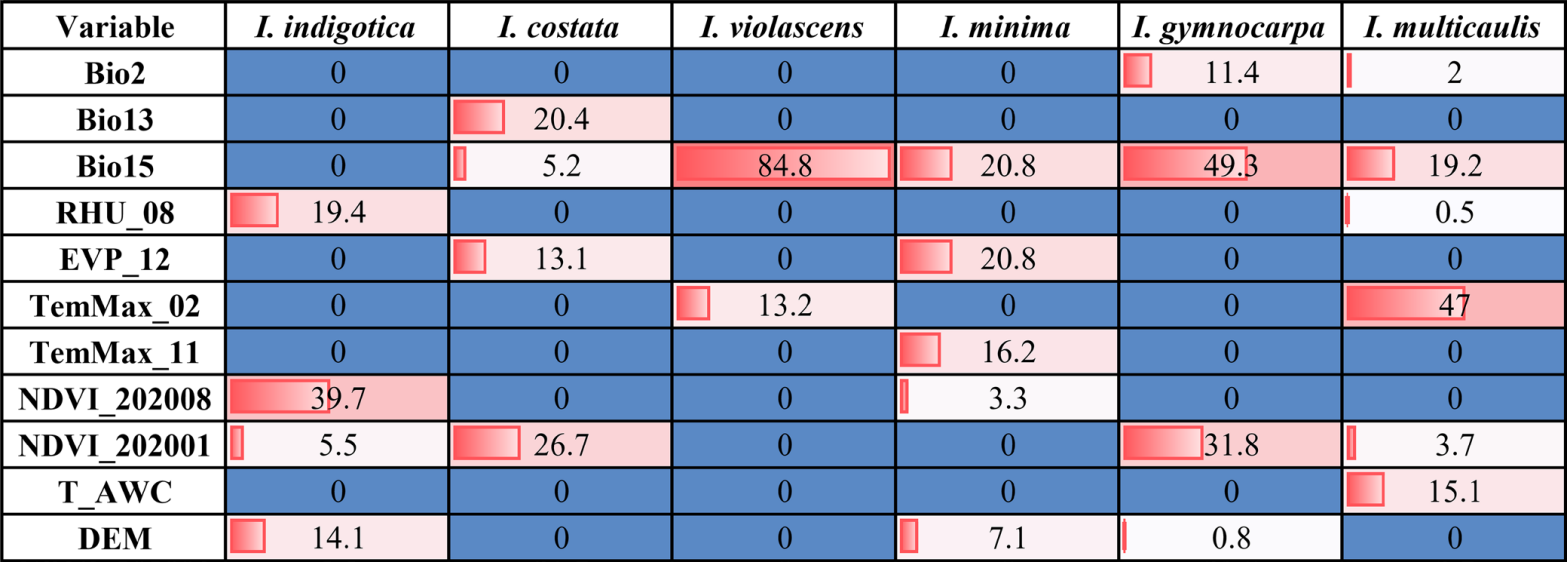
Permutation importance (%) of core environmental drivers for the six *Isatis* species. The results are based on permutation importance analysis from the MaxEnt models. Only variables that achieved an importance ≥ 10% in at least one species are shown. The color gradient from blue to red indicates low to high importance.

The jackknife test revealed significant differences in the key drivers among species (Figure 3), allowing for their classification into distinct ecological strategy types:

1. *Climate‐dominated*: The distributions of *I. violascens*, *I. minima* , *I. gymnocarpa*, and *I. multicaulis* were driven primarily by broad‐scale climatic factors, with precipitation seasonality (Bio15) being particularly influential (e.g., it contributed 84.8% of the permutation importance for *I. violascens*).
2. *Habitat indicator*: The distribution of *I. indigotica* was driven mainly by the August NDVI (NDVI_202008, 39.7%) and August relative humidity (RHU_08, 19.4%), whereas that of *I. costata* was most strongly associated with the precipitation of the wettest month (Bio13, 20.4%) and the January NDVI (NDVI_202001, 26.7%). Notably, the variable “proportion of irrigated cropland”, introduced specifically to assess the impact of cultivation, showed very low importance (< 1%) in the *I. indigotica* model and was not a core limiting factor.
3. *Species‐specific constraints*: The analysis also revealed several key environmental factors unique to particular species. For instance, the February maximum temperature (TemMax_02) was the most important driver for *I. multicaulis*, with a permutation importance of 47.0%.

**FIGURE 4:**
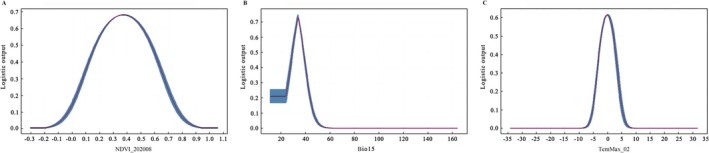
Response curves of habitat suitability to key environmental variables for representative *Isatis* species. (A) Response of *I. indigotica* suitability probability to the August NDVI (NDVI_202008). (B) Response of *I. violascens* suitability probability to precipitation seasonality (Bio15). (C) Response of *I. multicaulis* suitability probability to the February maximum temperature (TemMax_02).

To gain deeper insights into how the core environmental drivers influence habitat suitability, we further analyzed their response curves (Figure 4). These curves revealed distinct adaptation patterns corresponding to different ecological strategies.

1. *Indication of vegetation phenology*: The cultivated *I. indigotica* exhibited a unimodal response to the August NDVI (NDVI_202008) (Figure 4A). Its suitability probability peaked at an NDVI value of approximately 0.4, reflecting a strong association with areas of lush vegetation in the mid‐growing season (corresponding to agricultural landscapes). Suitability decreased outside this optimal range.
2. *Representative climate‐dominated species*: The habitat suitability probability for *I. violascens* decreased sharply with increasing precipitation seasonality (Bio15) (Figure 4B). When the value exceeded a certain threshold (approximately 65), the suitability probability consistently remained very low, indicating that a stable distribution of precipitation is a key limiting factor for its distribution.
3. *Temperature window constraint*: The response of *I. multicaulis* to the February maximum temperature (TemMax_02) showed a narrow optimal range (Figure 4C). The suitability probability peaked at approximately 10°C, whereas conditions below −5°C or above 20°C were unsuitable. These findings suggest that early spring temperature acts as a critical ecological filter for breaking dormancy and initiating growth.

### 3.3 Potential Distribution of *Isatis* Species Under Current Climate Conditions

**FIGURE 5:**
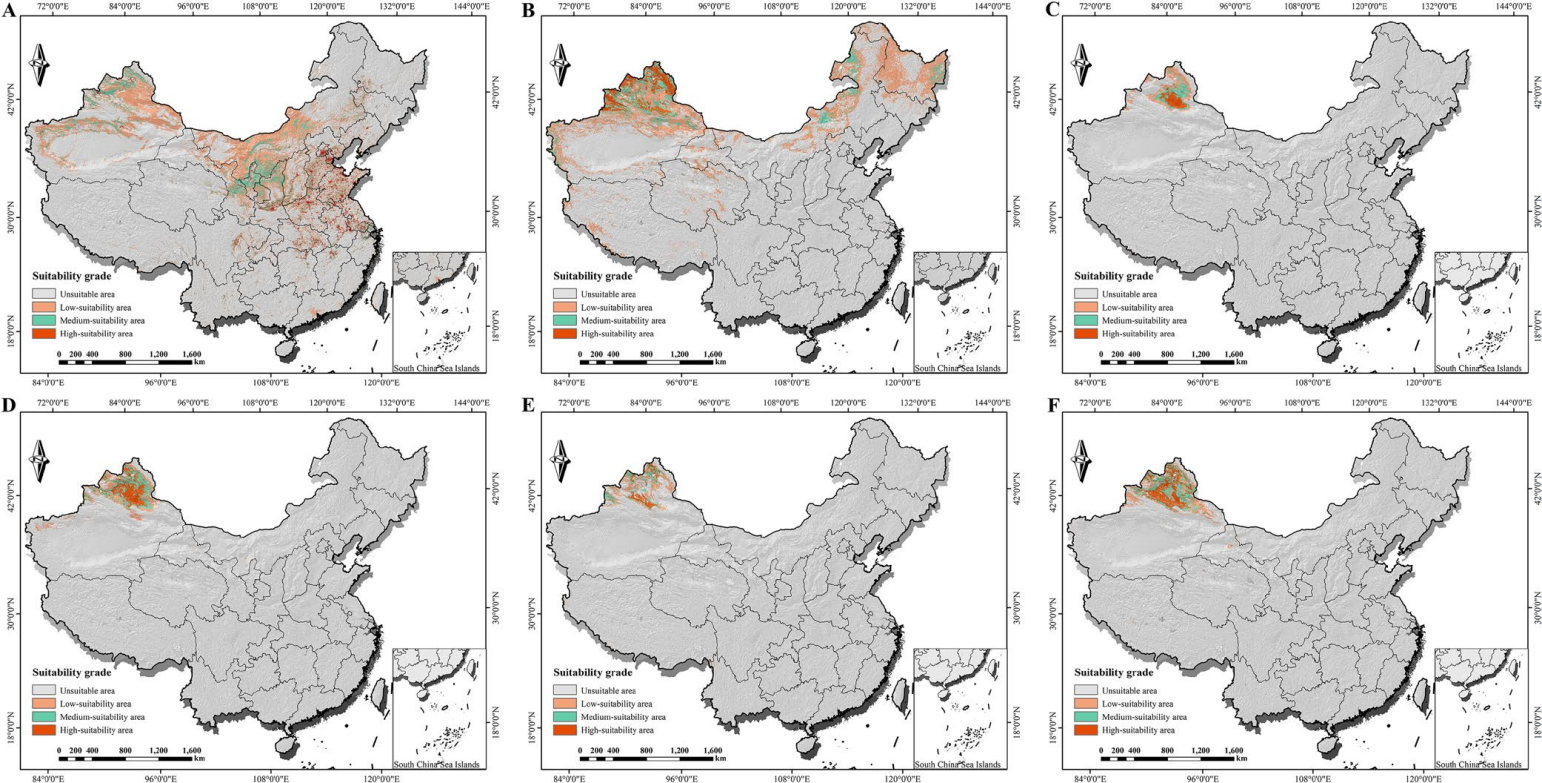
Predicted potential distribution of six *Isatis* species under current climatic conditions. Panels (A) to (F) represent *I. indigotica*, *I. costata* , *I. violascens*, *I. minima* , *I. gymnocarpa*, and *I. multicaulis*, respectively. Habitat suitability is classified into four grades (nonsuitable, low, medium, and high) using the natural breaks (Jenks) method. The basemap was obtained from the Standard Map Service of the Ministry of Natural Resources, China (Approval No.: GS (2023) 2767).

The optimized MaxEnt models predicted the potential geographical distributions of the six *Isatis* species under current climatic conditions (Figure 5). Overall, the suitable habitats exhibited distinct and contrasting geographical patterns. *I. indigotica* exhibited a vast, continuous suitable range covering China's major agricultural zones (e.g., North China Plain), reflecting its adaptation to farming landscapes. In contrast, *I. costata* showed a similarly extensive but fragmented distribution, concentrated in colder, high‐latitude montane regions (Altai, Tianshan, Greater Khingan Range), consistent with its preference for forest edges. The four other species—*I. violascens*, *I. minima* , *I. gymnocarpa*, and *I. multicaulis*—all displayed distribution patterns typical of arid and semiarid regions, with highly suitable habitats closely surrounding the peripheries of deserts in northwestern China, such as the Gurbantünggüt Desert.

Quantitative statistics of the suitable habitat areas further revealed substantial differences in range sizes among the species (Table 3). *I. indigotica* had the largest potential distribution area (196.67 × 10^4^ km^2^), whereas *I. gymnocarpa* possessed the most restricted range (19.95 × 10^4^ km^2^). The total suitable area for *I. costata* (163.14 × 10^4^ km^2^) was comparable to that of *I. indigotica*, but as described above, its spatial configuration was markedly more fragmented.

**TABLE 3:** Areal statistics of suitable habitats for *Isatis* species under current climate conditions.

Species	Unsuitable area	Low suitability	Medium suitability	High suitability	Total suitable area
*I. indigotica*	763.32	138.59	44.80	13.28	196.67
*I. costata*	796.87	118.43	29.43	15.28	163.14
*I. violascens*	940.31	9.94	5.78	3.97	19.69
*I. minima*	926.99	12.68	10.86	9.47	33.01
*I. gymnocarpa*	940.05	11.00	5.56	3.39	19.95
*I. multicaulis*	923.10	13.68	12.43	10.78	36.89

*Note:* Total suitable area = low suitability + medium suitability + high suitability. All area values are in units of ×10^4^ km^2^.

### 3.4 Potential Distribution Shifts of *Isatis* Species Under Future Climate Change

**FIGURE 6:**
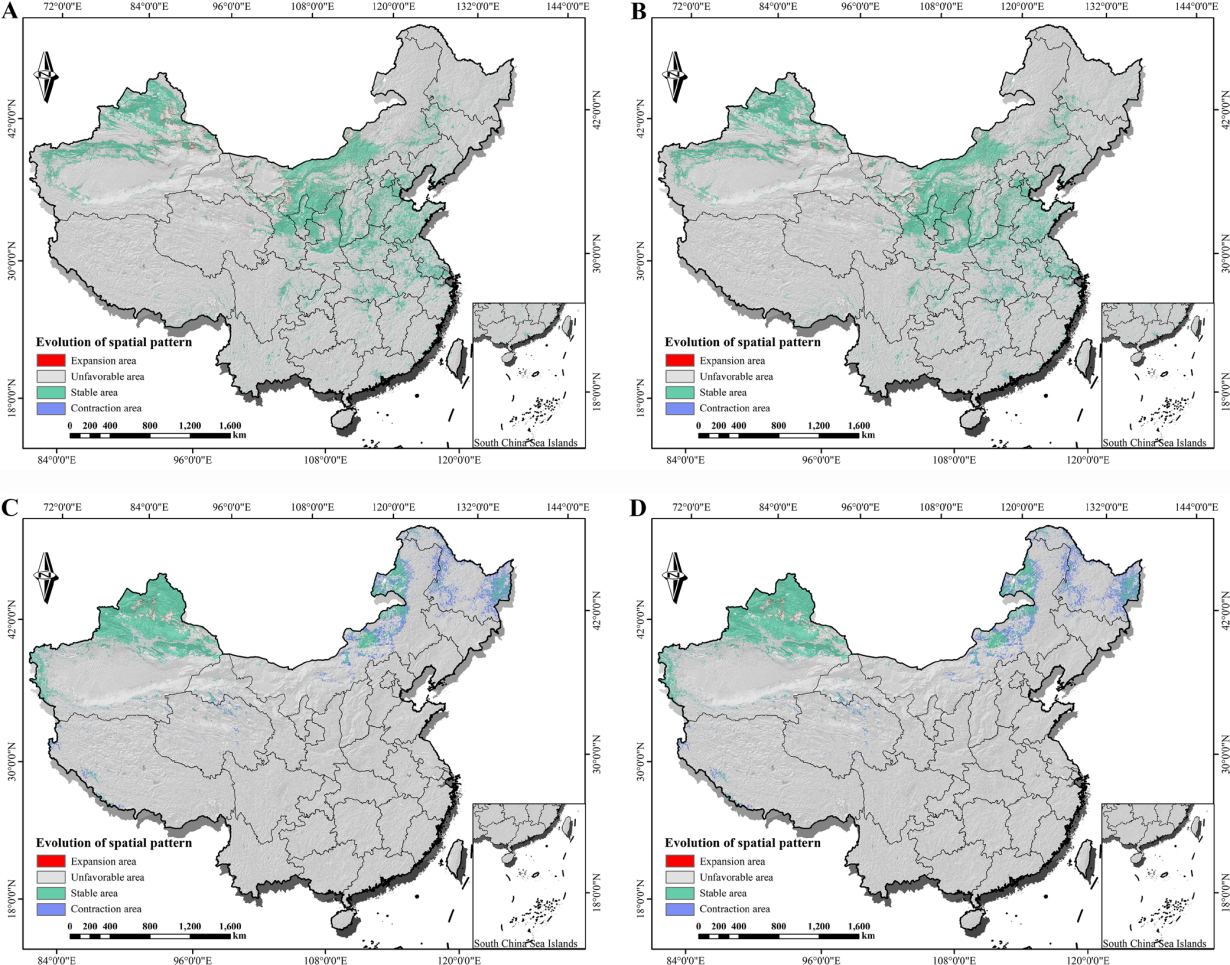
Spatial dynamics of suitable habitats for *I. indigotica* and *I. costata* under future climate change scenarios. Projections for the period 2041–2060 are shown for the two climate scenarios. (A) *I. indigotica* under SSP245, (B) *I. indigotica* under SSP585, (C) *I. costata* under SSP245, and (D) *I. costata* under SSP585. In all panels, ‘Stable’ indicates areas suitable under both current and future conditions, ‘Loss’ indicates areas suitable only under current conditions, and ‘Gain’ indicates areas suitable only under future conditions.

On the basis of future climate scenarios, we projected the potential geographical distributions for all six *Isatis* species (see complete suitability maps in Figures S3 and S4). As described in Section 3.2, the ecological strategies of these species differ significantly. To delve deeper into how this differentiation influences their responses to climate change, this section focuses on comparing the spatiotemporal shifts in suitable habitats between cultivated *I. indigotica* and its wild relative *I. costata* . Projections under future climate scenarios reveal starkly contrasting trajectories for the two species (Figure 6): the cultivated *I. indigotica* exhibited notable stability and resilience, whereas the wild *I. costata* faced a severe risk of range contraction.

Under both the SSP245 scenario and the SSP585 scenario, the distribution of *I. indigotica* demonstrated high stability coupled with limited northwest expansion (Figure 6, Table 4). The vast majority of its current suitable area (over 137 × 10^4^ km^2^ in both scenarios) was projected to persist as stable habitat, while the area of habitat loss was minimal (SSP245: 1.25 × 10^4^ km^2^; SSP585: 2.03 × 10^4^ km^2^). Concurrently, the models predicted the emergence of new suitable areas in northern Xinjiang. This pattern of “high stability, low loss, and limited expansion” resulted in a slight net increase in the total suitable area for *I. indigotica* (SSP245: +1.81%; SSP585: +0.81%).

**TABLE 4:** Changes in suitable habitat area for *I. indigotica* and *I. costata* under future climate scenarios.

Species	Scenario	Current suitable area	Stable area	Loss area	Gain area	Net change	Change rate
*I. indigotica*	SSP245	200.6	137.79	1.25	4.89	+3.64	+1.81%
	SSP585	198.55	137.01	2.03	3.63	+1.60	+0.81%
*I. costata*	SSP245	115.28	67.04	21.88	0.60	−21.28	−18.46%
	SSP585	115.32	67.08	21.91	0.57	−21.34	−18.51%

*Note:* The change rate was calculated as (net change/current suitable area) × 100%. All area values are in units of ×10^4^ km^2^.

In contrast, the suitable habitat for *I. costata* was projected to undergo substantial contraction (Figure 6, Table 4). Its shift pattern was characterized by the retention of core distribution areas but extensive loss in marginal zones, with almost no compensating range expansion. The spatial pattern clearly shows that these losses were primarily concentrated in the northeastern part of its current distribution (Figure 6). Consequently, the total suitable habitat area for *I. costata* was projected to decrease sharply by approximately 18.5% (a net loss of approximately 21.3 × 10^4^ km^2^), with a similar magnitude of loss under both climate scenarios.

These results clearly demonstrate that despite both being “habitat‐indicator” species, cultivated *I. indigotica* exhibits stronger resistance and adaptability to future climate change, whereas its wild relative *I. costata* is highly vulnerable, with its survival space likely to be severely diminished by climate change.

### 3.5 Niche Evolution Patterns of *Isatis* Species

**FIGURE 7:**
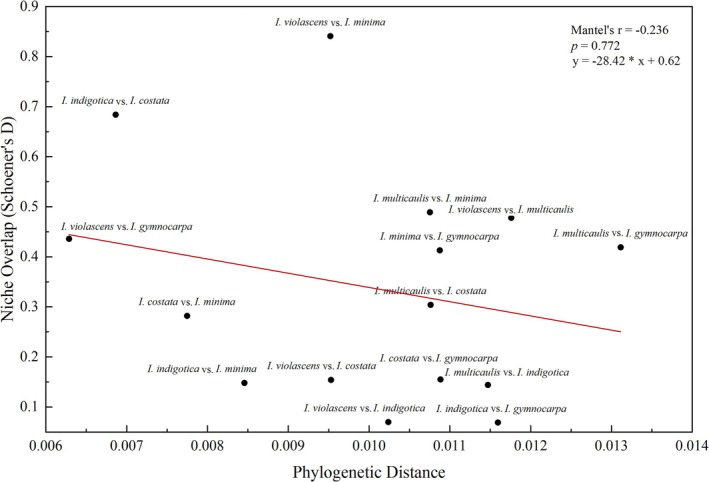
Relationships between phylogenetic distance and niche overlap among the six Isatis species. Each data point represents a pairwise comparison between two species (*n* = 15 pairs). The solid line indicates the linear regression trend. The Mantel test results revealed no significant correlation between phylogenetic distance and niche overlap (*r* = −0.236, *p* = 0.772).

To test the niche conservatism hypothesis within the genus *Isatis*, we analyzed the relationship between phylogenetic distance and niche overlap. The Mantel test revealed no significant correlation between phylogenetic distance and niche overlap among the *Isatis* species (*r* = −0.236, *p* = 0.772) (Figure 7). Examination of specific species pairs revealed diverse combinations of phylogenetic distance and niche overlap: the closely related species pair *I. indigotica–I. costata* (distance ~0.007) exhibited high niche overlap (Schoener's *D* = 0.684). In contrast, the more distantly related species pair *I. violascens–I. minima* (distance ~0.010) also showed high niche overlap (Schoener's *D* = 0.841). The relationships between phylogenetic distance and niche overlap varied even more among pairs of other species.

### 3.6 Species Richness Patterns and Biogeographic Hotspots

**FIGURE 8:**
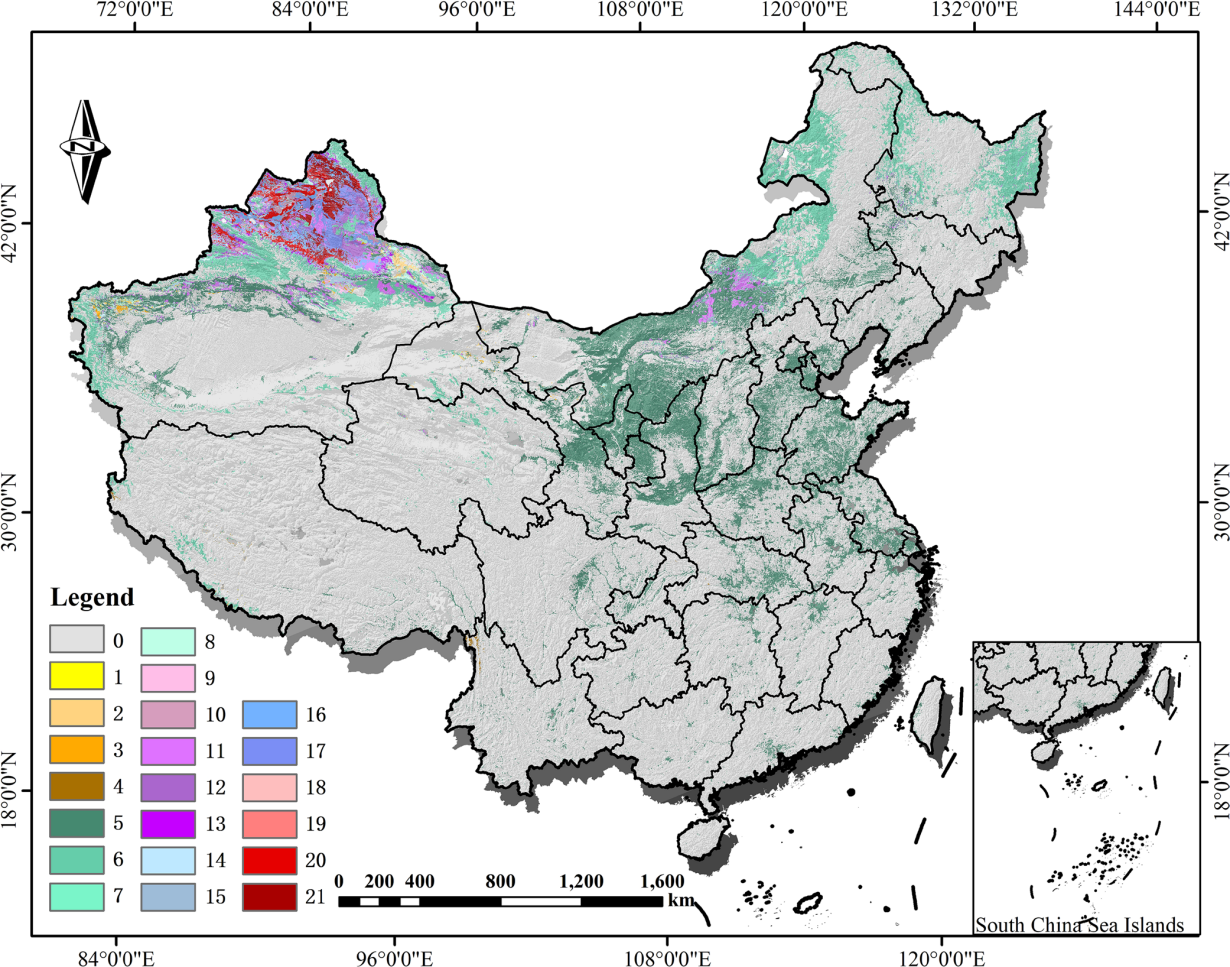
Species richness and co‐occurrence patterns of the six *Isatis* species in China. The map was generated by overlaying the binarized suitable habitat maps of all six species. Color codes from 0 to 21 represent different combinations of species, ranging from unsuitable habitat (0) to areas suitable for multiple species. A detailed legend explaining all species combinations is provided in Table S5.

Species richness maps were generated on the basis of the suitable binarized habitats of the six *Isatis* species to identify the biogeographic hotspots of the genus in China (Figure 8). Overall, the species richness of *Isatis* exhibited a macroscale pattern of “high in the northwest and low in the southeast”. Diversity hotspots (i.e., areas with ≥ 3 species) were primarily located in northwestern China, representing zones where the distribution ranges of wild relatives such as *I. violascens*, *I. minima* , *I. gymnocarpa*, and *I. multicaulis* overlap and converge. To depict the specific species combinations constituting these hotspots, the map uses a color code (0–21), where each value denotes a unique species assemblage, with the complete legend provided in Table S5.

**FIGURE 9:**
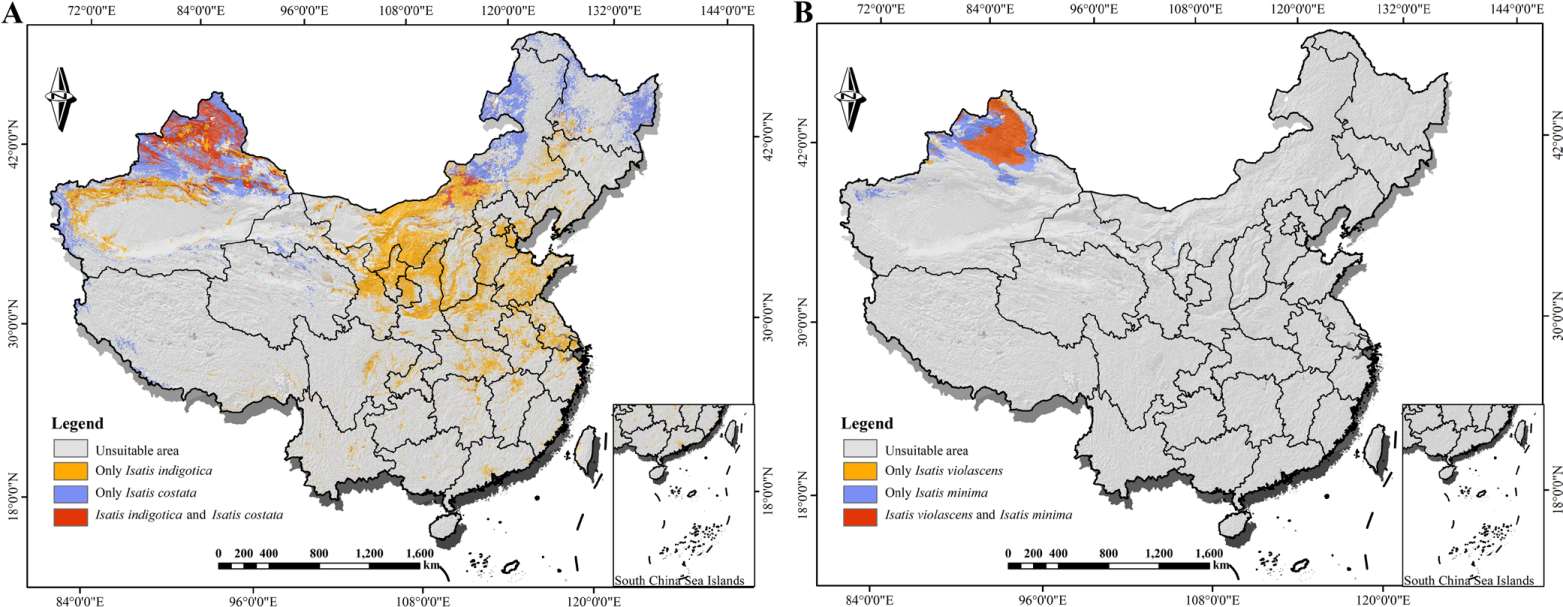
Comparison of species richness patterns between different ecological strategy groups. (A) Richness of the *I. indigotica–I. costata* group (cultivated and a wild relative with a habitat‐indicator strategy). (B) Richness of the *I. violascens–I. minima* group (wild species with a climate‐dominated strategy).

To investigate the distribution patterns of groups with different ecological strategies, we further compared the richness patterns of specific species groups (Figure 9). The “*I. indigotica*– *I.* costata” group (Figure 9A) exhibited a “northeast–northwest” dual‐core hotspot pattern. One hotspot center was located in the transition zone between the Northeast Plain and the North China Plain, and the other was located north of the Tianshan Mountains. This pattern reflects the overlay of the distribution of cultivated *I. indigotica* (widespread suitable habitat) and wild *I. costata* (fragmented and north‐shifted suitable habitat). In contrast, the richness hotspot of the “*I. violascens*– *I.* minima” group (Figure 9B) was highly concentrated in northwestern China, particularly surrounding the Gurbantünggüt Desert. Its high‐richness areas closely coincided with the major hotspot in the overall pattern but were more spatially constrained.

These comparative analyses of species groups demonstrate that although the overall diversity center of the genus is in the northwest, the species group containing the cultivated taxon has a significantly eastwards‐expanded distribution, reaching the core agricultural regions of eastern China. In contrast, the typical wild species group is strictly confined to the natural habitats of the northwest.

## 4 Discussion

### 4.1 Drivers of Ecological Strategy Differentiation in *Isatis*

The contrasting environmental drivers shaping *Isatis* distributions reflect divergent life‐history strategies. Annual ephemerals (e.g., *I. violascens* and *I. minima* ) are strongly constrained by precipitation seasonality (Bio15), a key adaptation to unpredictable rainfall in arid regions. These plants exhibit a typical “bet‐hedging” strategy (Venable 2007; Gremer and Venable 2014), synchronizing their life cycles with brief wet periods to complete reproduction before drought onset. Intra‐annual precipitation distribution thus acts as a critical environmental filter, more so than total annual precipitation (Lu et al. 2016).

Biennial species show distinct patterns. Cultivated *I. indigotica* is closely associated with August NDVI (NDVI_202008), indicating its niche is embedded within agricultural ecosystems. Domestication has decoupled its distribution from direct climatic limitations, aligning it instead with farmland productivity indicators (Weiss et al. 2020; Joshi et al. 2023). In contrast, wild *I. costata* depends on wettest‐month precipitation (Bio13) and January NDVI—likely reflecting preferences for forest‐edge habitats with stable water availability and protective snow cover that supplies meltwater in spring (De Frenne et al. 2021). This requirement for stable hydrothermal conditions fundamentally differs from the rainfall‐tracking strategy of annual ephemerals and the adaptation of cultivated species to managed environments.

These divergent ecological strategies reflect evolutionary adaptation to heterogeneous habitats—from arid deserts to farmlands and montane forest edges. *The findings align with life‐history theory*: annuals track interannual moisture variability, while biennials are more constrained by thermal regimes and habitat stability, providing empirical support for life‐history strategy as a predictor of environmental filtering axes.

### 4.2 Complex Niche Evolution Patterns in *Isatis*

The Mantel test revealed no significant correlation between phylogenetic distance and niche overlap among *Isatis* species (*r* = −0.236, *p* = 0.772), rejecting strict phylogenetic niche conservatism at the genus level (Kim et al. 2025; Padilha et al. 2025). However, in‐depth analysis of specific species pairs revealed a more complex evolutionary picture than the overall pattern—both conservative evolution and convergent evolution coexist within the genus.

Signals of niche conservatism were detected in some closely related species. For instance, the closely related *I. indigotica* and *I. costata* (branch length distance ~0.007) exhibited high niche overlap (Schoener's *D* = 0.684). This aligns with the expectation of niche conservatism, potentially stemming from their recent divergence from a common ancestor without subsequent significant niche differentiation, thus retaining similar physiological tolerances and ecological preferences for forest‐edge and anthropogenically influenced habitats. However, a more insightful finding is the niche convergence observed between distantly related species. Despite their relatively greater phylogenetic distance (branch length distance ~0.010), *I. violascens* and *I. minima* showed high niche overlap (Schoener's *D* = 0.841). This pattern of distant relationship but high niche similarity strongly suggests convergent evolution (Hufford et al. 2019). We infer that, although these two species belong to different lineages in the phylogenetic tree, they have independently evolved similar life‐history strategies (annual ephemeral) and analogous adaptations to key environmental factors, such as high precipitation seasonality, in response to the extremely arid desert environment of northwestern China.

This complexity demonstrates that niche conservatism is not universal (Mittelbach and Schemske 2015); in heterogeneous environments, contemporary natural selection can override phylogenetic constraints. The coexistence of conservatism and convergence exemplifies the dynamic interplay between historical legacy and contemporary adaptation. Notably, this pattern aligns closely with life‐history strategy: convergence occurs in annual ephemerals ( *I. minima* and *I. violascens*), while conservatism characterizes the biennial pair ( *I. costata* and *I. indigotica*), suggesting that life‐history strategy may influence the direction of niche evolution—a hypothesis warranting further investigation.

### 4.3 Divergent Future Fates and Underlying Causes for *Isatis*

Building on the ecological and evolutionary differences above, our models project starkly contrasting futures for cultivated *I. indigotica* and its wild relative *I. costata* —the former remains stable, while the latter loses ~18.5% of its suitable range.

The stability of cultivated *I. indigotica* largely stems from its deep integration within agricultural ecosystems. Extensive human cultivation and ongoing germplasm resource management collectively create a substantial “human‐mediated buffer,” effectively cushioning the direct impacts of climate change. This buffering effect is reflected in its niche association with August NDVI—an integrated indicator of agricultural productivity—rather than direct climatic constraints, suggesting that management practices (including irrigation) have aligned its ecological requirements with the productivity patterns of farmlands. Agricultural management interventions can significantly increase adaptive capacity to climate change (Liaqat et al. 2024; Xiong et al. 2024). Compared with their wild counterparts, cultivated populations often consequently exhibit greater climate resilience (Shukla et al. 2024), a phenomenon widely documented in the adaptation strategies of various crops (Yan et al. 2022). The stability of *I. indigotica* under future scenarios likely reflects this buffering effect of agricultural management, which mitigates climatic stress—a benefit not available to its wild relative *I. costata* .

In sharp contrast, the vulnerability of *I. costata* is an inevitable consequence of its wild ecological strategy and specific habitat requirements. Our study revealed that its distribution is strictly constrained by the precipitation of the wettest month and the winter NDVI. This narrow niche breadth, coupled with its obligate dependence on relatively fragmented habitats such as forest edges and mountains, renders its populations inherently precarious, existing in a fragile equilibrium (Slatyer et al. 2013). Climate change not only directly alters its suitable hydrothermal niche but also likely exacerbates the fragmentation of its existing habitats, hindering its ability to track suitable environments through natural dispersal (Jin et al. 2020). The fate of *I. costata* typifies the severe challenges faced by nondomesticated, ecologically specialized wild species in the face of rapid climate change, and serves as an early warning for the conservation of wild *Isatis* relatives.

### 4.4 Conservation Implications and Recommendations for *Isatis*

None of the six Chinese *Isatis* species are listed in national or international protection frameworks, nor have they been IUCN‐evaluated (National Forestry and Grassland Administration M O A 2021; CITES, n.d.). However, this absence reflects assessment gaps rather than low risk—congeners such as *Isatis karjaginii* Schischk. (Endangered) and *Isatis platyloba* Link ex Steud. (Vulnerable) appear on the IUCN Red List (IUCN, n.d.), indicating conservation concern within the genus.

Our projections provide the first quantitative evidence of climate risk: *I. costata* is projected to lose ~18.5% of its suitable habitat, despite lacking protected status. This underscores the urgency of formal assessments and a differentiated framework prioritizing wild relatives over cultivated species. Unlike *I. indigotica*, which benefits from agricultural buffering, wild relatives like *I. costata* harbor irreplaceable evolutionary heritage and genetic diversity crucial for future adaptation; their loss would be irreversible (Ortiz et al. 2024; Mathan et al. 2025). We recommend: (1) prioritizing *I. costata* population assessments; (2) establishing in situ protection networks; (3) implementing ex situ conservation; and (4) strengthening long‐term monitoring.

Several limitations of this study should be acknowledged. First, the sample sizes for four wild species were modest, which may increase prediction uncertainty despite high AUC values. While spatial rarefaction and default MaxEnt parameters were applied to minimize overfitting, small sample sizes remain a constraint. Second, our irrigation variable did not capture fine‐scale variation in human impacts, likely due to near‐ubiquitous irrigation across cultivation areas. Future studies incorporating additional field surveys across broader geographic ranges, complementary data on farming practices, and multiple climate models would help validate and refine our projections.

## 5 Conclusion

This study demonstrated that the distribution, niche evolution, and response to future climate change in the genus *Isatis* are shaped by the interplay between life‐history strategies and phylogenetic history.

Key findings include (1) a divergence in ecological strategies, where annual ephemerals rely on climatic fluctuations, which is indicative of an opportunistic strategy, while biennial species are linked to vegetation phenology markers of habitat stability; (2) a complex pattern of niche evolution, with evidence for both niche conservatism in closely related species and niche convergence in distantly related species, underscoring the power of natural selection over phylogenetic constraints in heterogeneous environments; and (3) a stark divergence in future trajectories, where the cultivated species exhibits resilience due to its integration into agricultural systems, in sharp contrast to its wild relative, which faces severe range contraction due to its narrow niche and habitat fragmentation.

Our findings underscore the critical need to distinguish between cultivated and wild germplasm for medicinal plant conservation. Despite their less apparent immediate economic value, wild relatives constitute an irreplaceable evolutionary heritage and a vital genetic reservoir for future adaptation and thus must be prioritized in conservation strategies.

## Author Contributions

**Min Wei:** data curation (equal), formal analysis (equal), resources (equal), writing – original draft (equal). **Yong Su:** conceptualization (lead). **Hongzhuan Shi:** writing – review and editing (lead). **Tao Bao:** conceptualization (supporting). **Qiaosheng Guo:** writing – review and editing (equal).

## Funding

This work was supported by the National Key Research and Development Program of China (Project Title: Spatiotemporal Analysis of the Quality Formation of Chinese Herbal Medicines and Demonstration of Pseudocultivation Research; Special Program: Modernization of Traditional Chinese Medicine; Project No.: 2023YFC3503800).

## Ethics Statement

In this study, ecological modeling analysis is performed on the basis of public data and nondestructive field surveys. All species occurrence data were obtained in compliance with ethical guidelines: (1) A portion of the data originated from field surveys conducted by the authors in China from 2021 to 2023. These surveys involved only photographing and recording the GPS locations of plants, ensuring that there was no impact on wild populations or their habitats. (2) The remaining data were sourced from public databases, including the CVH and the GBIF. The access to and use of these data complied with the respective platform user agreements. None of the species investigated in this study are listed on the Red List of Endangered Species in China. All research activities adhered to relevant institutional, national, and international guidelines and legislation.

## Consent

The authors have nothing to report.

## Conflicts of Interest

The authors declare no conflicts of interest.

## Supporting information


**Figure S1:** Pairwise Pearson correlation matrices of the environmental variables retained after the three‐step screening procedure for each *Isatis* species. Panels (A) to (F) represent *I. indigotica*, *I. costata* , *I. violascens*, *I. minima*, *I. gymnocarpa*, and *I. multicaulis*, respectively. The final variable sets for each species are listed in Table S3.

**Figure S2:** Phylogenetic tree of the six studied *Isatis* species inferred from complete chloroplast genomes. The tree was reconstructed using the maximum likelihood method. The numbers at the nodes represent bootstrap support values from 1000 replicates. The scale bar indicates the number of substitutions per site.

**Figure S3:** Predicted potential distributions of six *Isatis* species under the future SSP245 scenario (2041–2060). Panels (A) to (F) represent *I. indigotica*, *I. costata* , *I. violascens*, *I. minima* , *I. gymnocarpa*, and *I. multicaulis*, respectively. Habitat suitability is classified into four grades (nonsuitable, low, medium, and high) using the natural breaks method.

**Figure S4:** Predicted potential distributions of six *Isatis* species under the future SSP585 scenario (2041–2060). Panels (A) to (F) represent *I. indigotica*, *I. costata* , *I. violascens*, *I. minima* , *I. gymnocarpa*, and *I. multicaulis*, respectively. Habitat suitability is classified into four grades (nonsuitable, low, medium, and high) using the natural breaks method.

**Figure S5:** Field photographs of *Isatis* species in their representative habitats. (A) *I. costata* at forest edge, Tianshan Mountains, Xinjiang. (B) *I. violascens* growing on sand dunes, Junggar Basin, Xinjiang. (C) *I. minima* in desert steppe, Gurbantünggüt Desert. (D) *I. gymnocarpa* on gravel slopes, Xinjiang. (E) *I. multicaulis* in sandy habitat, Xinjiang. (F) Cultivated *I. indigotica* in agricultural fields.

**Table S1:** Occurrence records of *Isatis* species used in this study.

**Table S2:** Full list and descriptions of the 144 initial environmental variables.

**Table S3:** Percent contribution and permutation importance of the final environmental variables in the MaxEnt models for each *Isatis* species.

**Table S4:** Pairwise matrices of phylogenetic distance and niche overlap (Schoener's *D*) for *Isatis* species pairs.

**Table S5:** Detailed legend for the species co‐occurrence map (Figure 8).

## Data Availability

All the data supporting the findings of this study are available within the article and its Supporting Information or have been deposited in public repositories. (1) *Species occurrence data*: The final, spatially rarefied dataset of species occurrence points (*n* = 229 records) used for modeling in this study is provided in the Table S1. (2) *Environmental variables*: Descriptions and sources for all 144 initial environmental variables are listed in Table S2. The specific variable sets used for the final model of each species, along with their percent contribution and permutation importance, are detailed in Table S3. (3) *Model outputs and niche analysis data*: The habitat suitability maps generated by the species distribution models, the phylogenetic tree file, the matrices used for the niche conservatism test, and all other supplementary figures (including the newly added Figure S5) and data have been deposited in the Figshare repository and are accessible via https://doi.org/10.6084/m9.figshare.30734258. (4) *Phylogenetic data*: The complete chloroplast genome sequences underlying the phylogenetic framework of this study are available from the NCBI GenBank database, with accession numbers provided in the Supporting Information.
