# Supplementary figures and images for: Phylogenetics, Niche Evolution, and Distribution Dynamics of Isatis Species Under Climate Change
Source: Ecol Evol. 2026 May 4;16(5):e73514. doi: 10.1002/ece3.73514 (PMC13139723; doi:10.1002/ece3.73514)

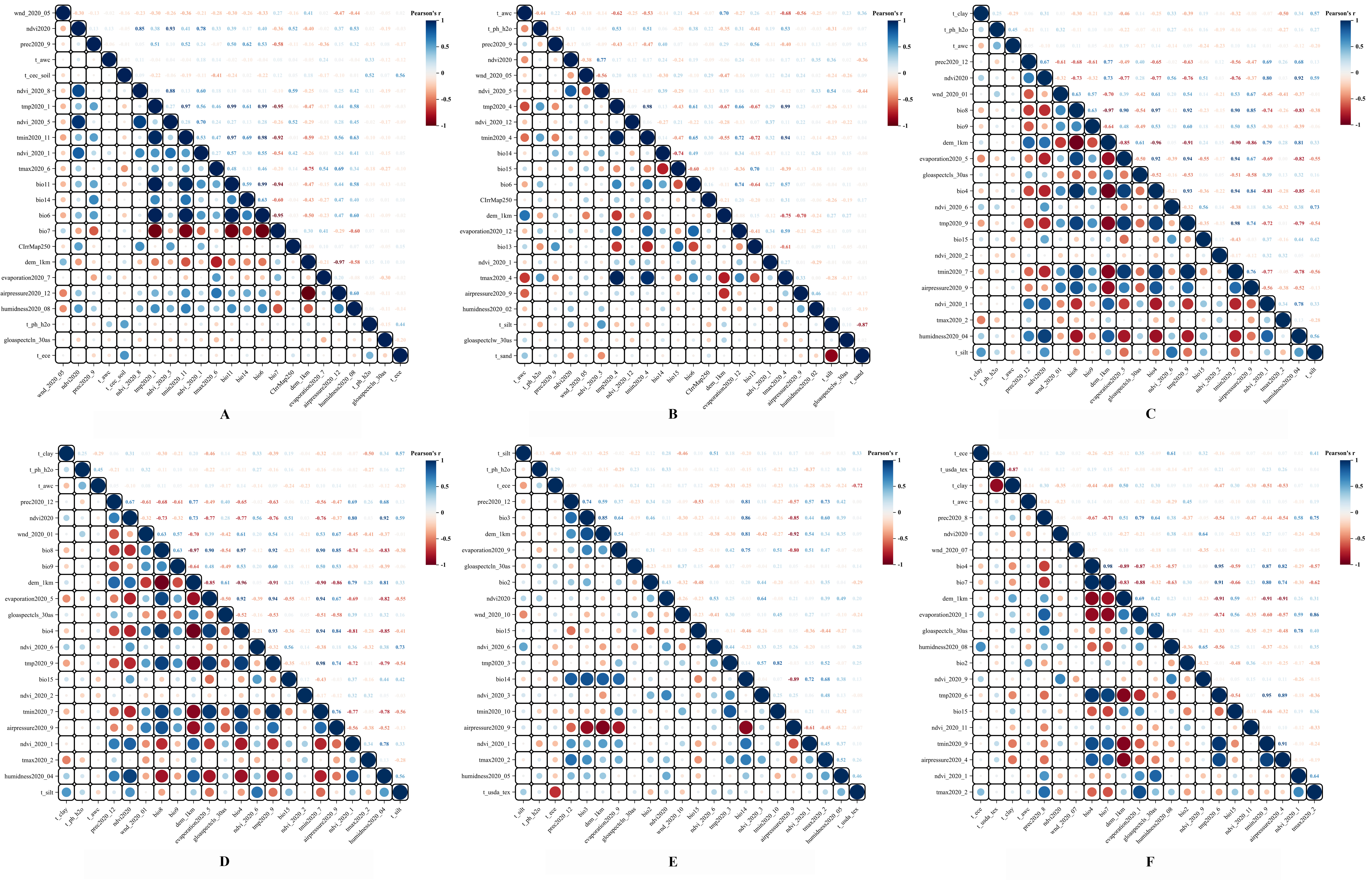

Supplement: Supplementary file 1 — Figure S1: Pairwise Pearson correlation matrices of the environmental variables retained after the three‐step screening procedure for each Isatis species. Panels (A) to (F) represent I. indigotica, I. costata , I. violascens, I. minima, I. gymnocarpa, and I. multicaulis, respectively. The final variable sets for each species are listed in Table S3. [file ECE3-16-e73514-s010.tif]

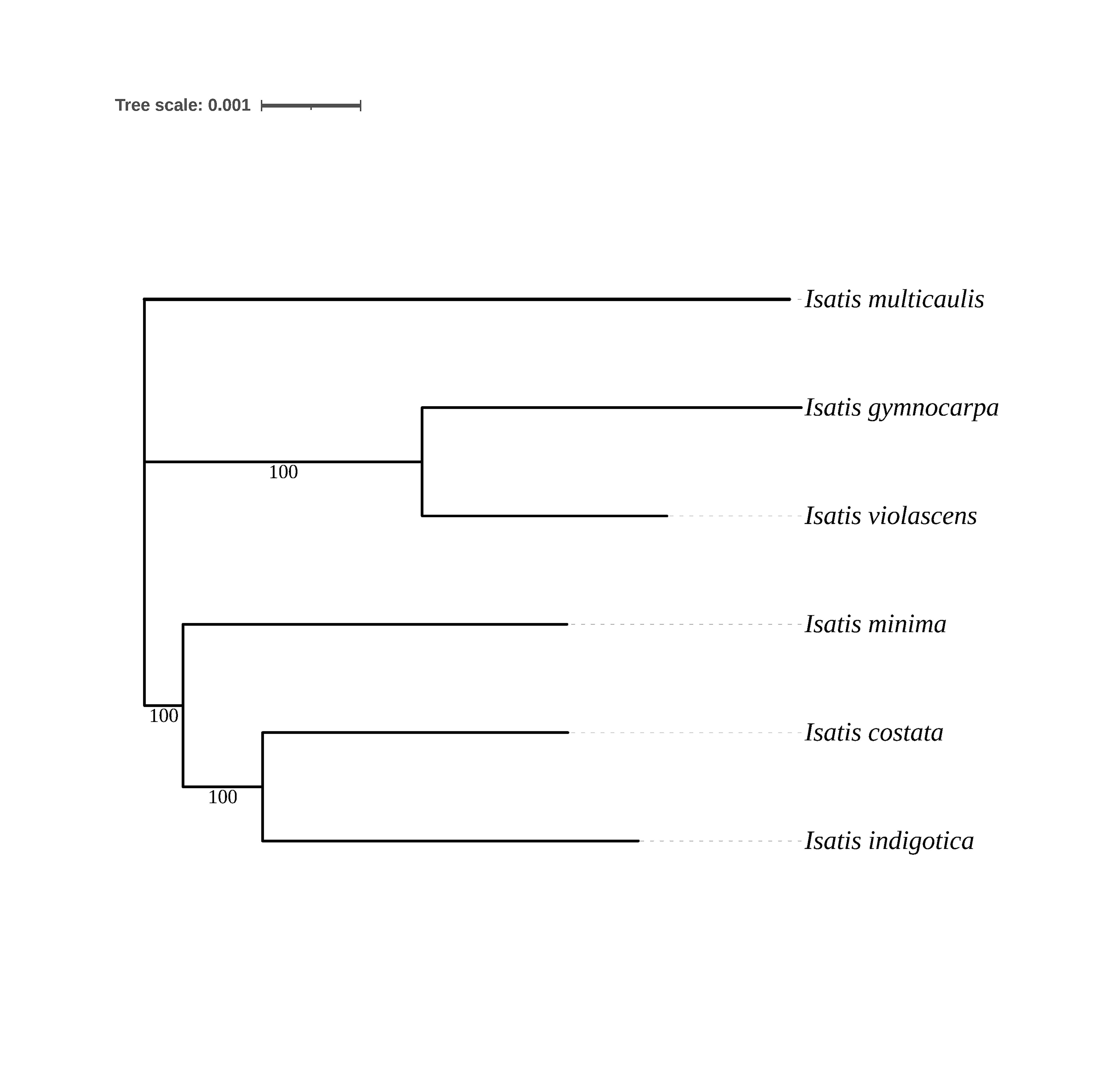

Supplement: Supplementary file 2 — Figure S2: Phylogenetic tree of the six studied Isatis species inferred from complete chloroplast genomes. The tree was reconstructed using the maximum likelihood method. The numbers at the nodes represent bootstrap support values from 1000 replicates. The scale bar indicates the number of substitutions per site. [file ECE3-16-e73514-s008.tif]

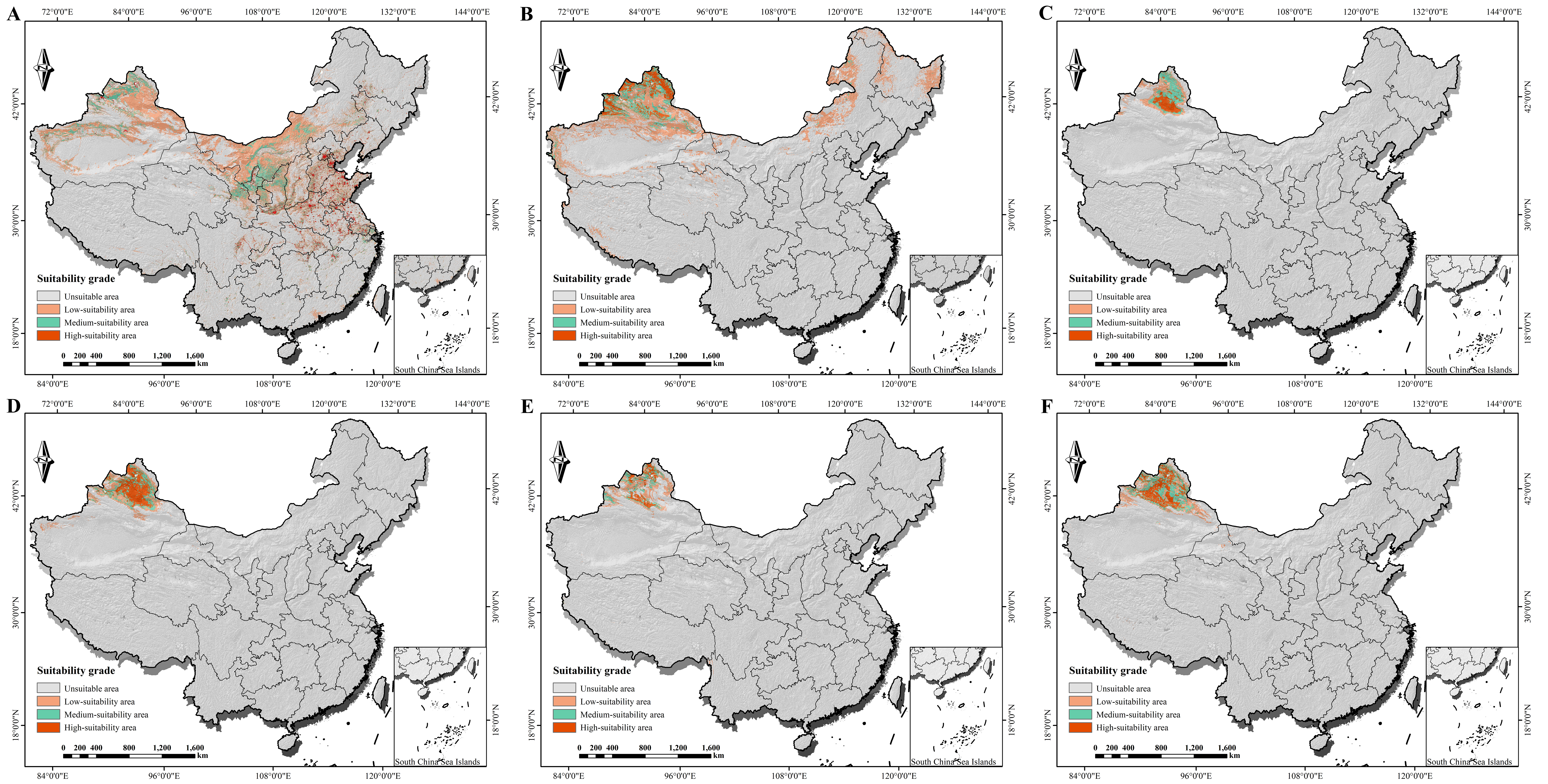

Supplement: Supplementary file 3 — Figure S3: Predicted potential distributions of six Isatis species under the future SSP245 scenario (2041–2060). Panels (A) to (F) represent I. indigotica, I. costata , I. violascens, I. minima , I. gymnocarpa, and I. multicaulis, respectively. Habitat suitability is classified into four grades (nonsuitable, low, medium, and high) using the natural breaks method. [file ECE3-16-e73514-s002.tif]

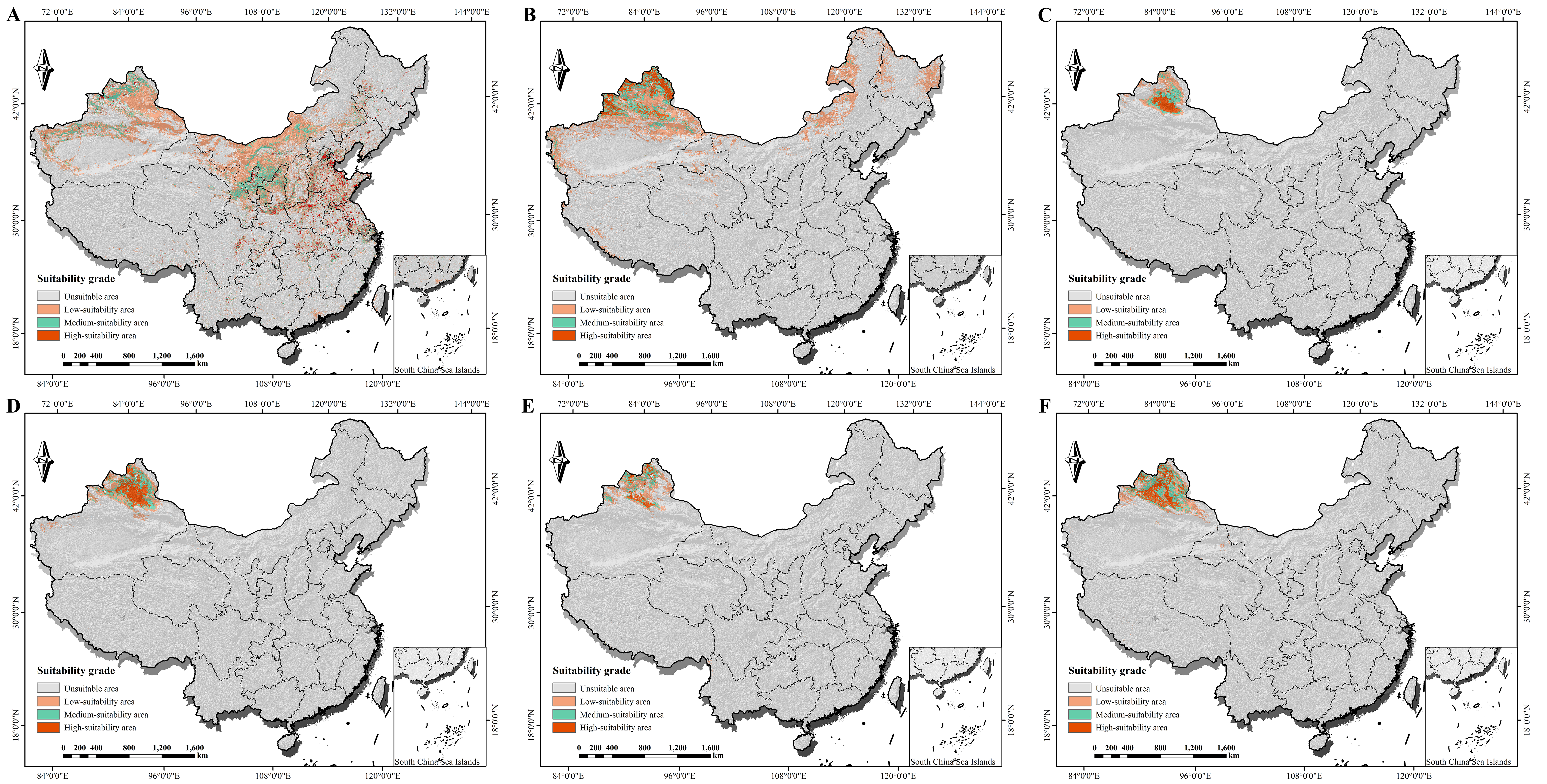

Supplement: Supplementary file 4 — Figure S4: Predicted potential distributions of six Isatis species under the future SSP585 scenario (2041–2060). Panels (A) to (F) represent I. indigotica, I. costata , I. violascens, I. minima , I. gymnocarpa, and I. multicaulis, respectively. Habitat suitability is classified into four grades (nonsuitable, low, medium, and high) using the natural breaks method. [file ECE3-16-e73514-s007.tif]

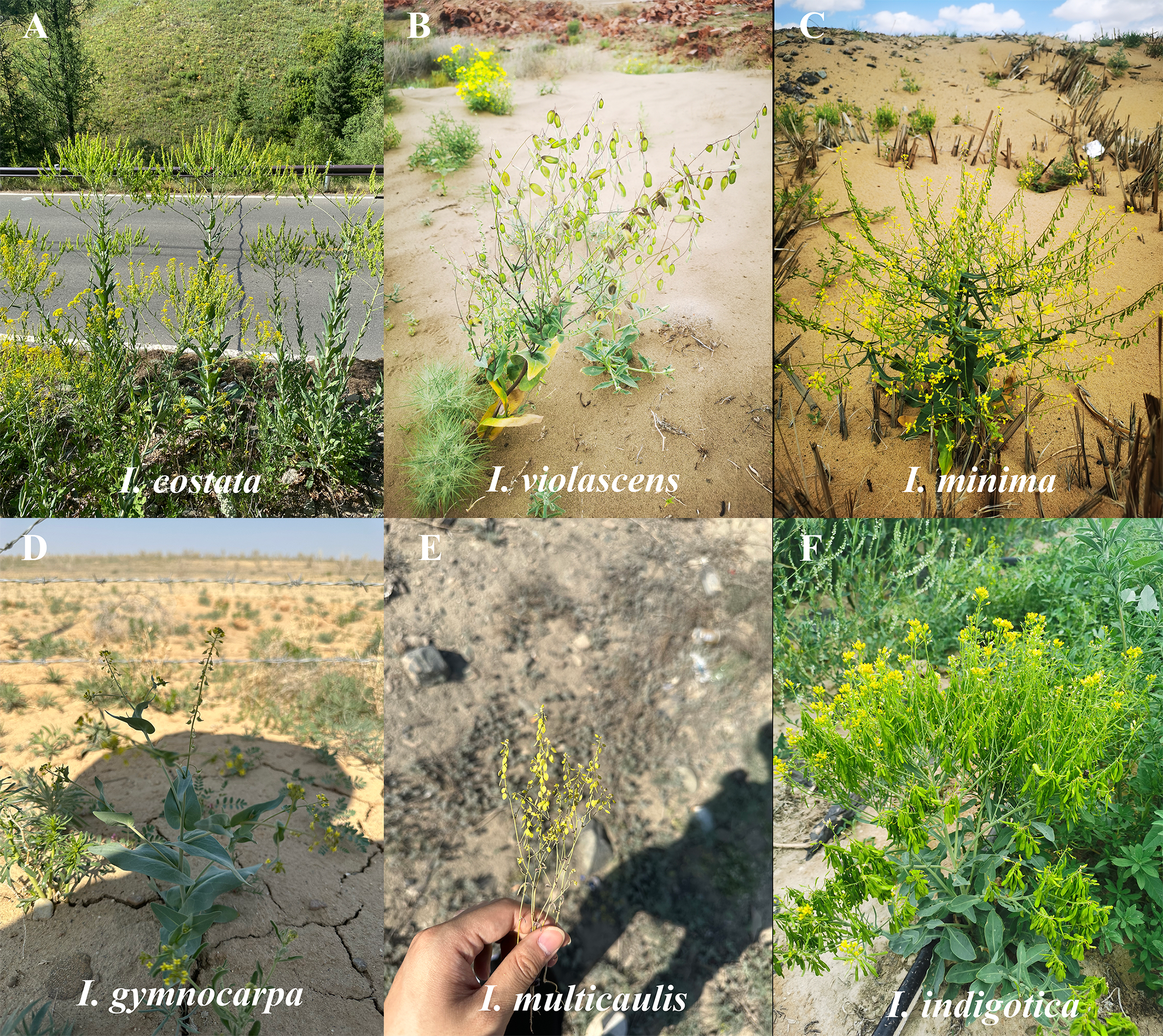

Supplement: Supplementary file 5 — Figure S5: Field photographs of Isatis species in their representative habitats. (A) I. costata at forest edge, Tianshan Mountains, Xinjiang. (B) I. violascens growing on sand dunes, Junggar Basin, Xinjiang. (C) I. minima in desert steppe, Gurbantünggüt Desert. (D) I. gymnocarpa on gravel slopes, Xinjiang. (E) I. multicaulis in sandy habitat, Xinjiang. (F) Cultivated I. indigotica in agricultural fields. [file ECE3-16-e73514-s004.tif]
